# Large retroperitoneal isolated fibrous cyst in absence of preceding trauma or acute pancreatitis

**DOI:** 10.1186/s12893-015-0016-2

**Published:** 2015-03-14

**Authors:** Julie Ahn, Manju D Chandrasegaram, Khaled Alsaleh, Benjamin L Woodham, Adrian Teo, Amithaba Das, Neil D Merrett, Christos Apostolou

**Affiliations:** Division of Surgery, University of Western Sydney, Sydney, Australia; Upper Gastrointestinal Unit, Bankstown Hospital, Sydney, Australia

**Keywords:** Retroperitoneal cyst, Fibrous cyst, Retroperitoneal mass, Pseudocyst, Cystic mass

## Abstract

**Background:**

Isolated retroperitoneal cystic masses are uncommon with an estimated incidence of 1/5750 to 1/250,000. The majority present with size related symptoms, complications, or a mass. Approximately a third of patients are asymptomatic and are diagnosed incidentally.

Aetiologies of retroperitoneal cystic masses (RPC) include mesenteric, omental, splenic and enteric duplication cysts. Neoplastic RPCs can be divided into epithelial (mucinous or serous cystadenoma), mesothelial (mesothelioma), germ cell (cystic teratoma) and cystic changes in a solid neoplasm (paraganglioma, neurilemmoma, sarcoma).

**Case presentation:**

A 53 year-old man presented to us with abdominal pain related to a large mass in his left upper quadrant with associated anorexia and weight loss. He gave no history of previous trauma and denied having symptoms or a history of pancreatitis. He said he had felt this mass increasing in size over the course of several years.

Clinical examination of his abdomen revealed a large firm left sided mass extending to his left upper quadrant.

Imaging with computed tomography (CT) and magnetic resonance imaging cholangio-pancreatogram (MRCP) revealed a 13.7 cm × 12.2 cm × 10.9 cm cystic lesion in the retroperitoneum which was separate from the kidney, pancreas, spleen and bowel. At laparotomy, this mass was easily dissected from the surrounding viscera and was excised completely intact.

Histopathological assessment found the mass to be a large fibrous pseudocyst with no epithelial lining.

**Conclusion:**

We present a rare case of an isolated large retroperitoneal fibrous pseudocyst unrelated to previous pancreatitis which was successfully managed with surgery.

## Background

Retroperitoneal cystic masses, which are isolated and separate from surrounding major organs are uncommon, with an estimated incidence of 1/5750 to 1/250,000 [1]. Approximately one third of patients with a retroperitoneal cyst (RPC) are asymptomatic and are diagnosed as an incidental finding [1,2]. Two thirds present with symptoms relating to size and complications, and this most often is from a noticeable abdominal mass [3].

The most common cause of retroperitoneal cystic masses are pseudocysts related to pancreatitis which occur more frequently with acute-on chronic pancreatitis [4]. Other causes of retroperitoneal cysts include cysts that develop from surrounding structures such as mesenteric, omental, splenic and enteric duplication cysts. Neoplastic causes included cystadenomas, mesotheliomas, and cystic degeneration that can arise from solid neoplasms [2]. Non-neoplastic causes include haematomas, urinomas, lymphoceles, pancreatic and non-pancreatic pseudocysts [2].

We report a case of a patient presenting with a large isolated retroperitoneal cystic mass in the absence of preceding trauma or pancreatitis.

## Case presentation

A 53-year-old man presented with a four-week history of worsening abdominal pain, anorexia and weight loss. This was on a background of a known large left sided cystic mass, which was discovered on imaging while he resided overseas nine months ago. He described his symptoms up until this presentation as mild but now complained of post-prandial abdominal pain, occasional vomiting and diarrhea. His impression was that the mass had been increasing in size for the past month. His past medical history included hepatitis C and diverticulosis. He gave no history of previous trauma and denied having symptoms or a history of pancreatitis in the past. There was no history of gallstones and no evidence of gallstones on prior or current imaging. His alcohol intake approximated 20 standard drinks a week. He also gave a 30 pack-year smoking history in addition to smoking marijuana daily.

On clinical examination, he had a grossly visible mass in his left upper quadrant, which could be appreciated at the end of the bed. This mass was firm and mildly tender. His blood examination revealed haemoglobin 124 g/L (Reference range: 130–180 g/L), l white cell count of 6.4 x10^9^/L (Reference range: 4–11 x10^9^/L) and CRP of 11.3 mg/L (Reference range: <5 mg/L). Renal function and liver enzymes were normal. His CEA was elevated at 9.5 μg/L (Reference range: <2.5 μg/L). CA 19.9 and AFP tumour marker were normal.

**Figure 1:**
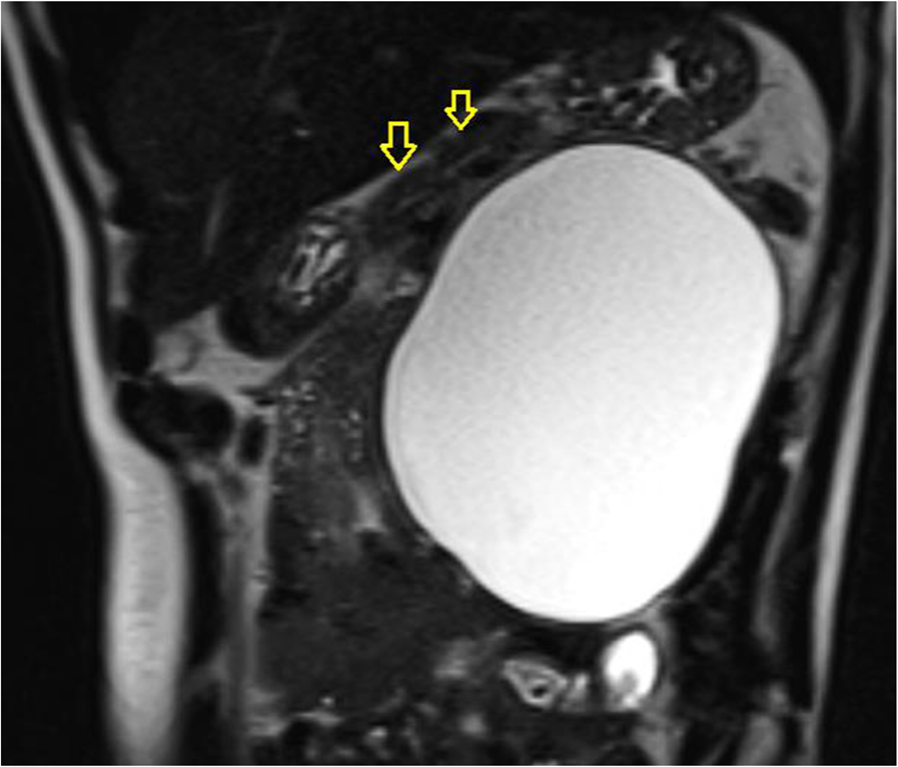
**Coronal MRI (T2 phase) showing large retroperitoneal cyst with no septations.** The retroperitoneal cyst is separate to the pancreas (Pancreas – yellow arrow).

Computed tomography (CT) scan of the abdomen and magnetic resonance cholangio-pancreatogram (MRCP) revealed a large retroperitoneal cystic lesion in the left flank with no septations or enhancement, measuring 13.7 cm craniocaudally, 12.2 cm transversely and 10.9 cm anteroposteriorly. It appeared to be isolated and separate from the adjacent organs including the pancreas, stomach, spleen and left kidney (Figure 1). There were two small simple cysts in the spleen. There was mild to moderate dilation of the left renal pelvis, calyces and proximal left ureter. This was likely due to partial mechanical distortion from the mass, which displaced the left ureter laterally. The right kidney was normal.

**Figure 2:**
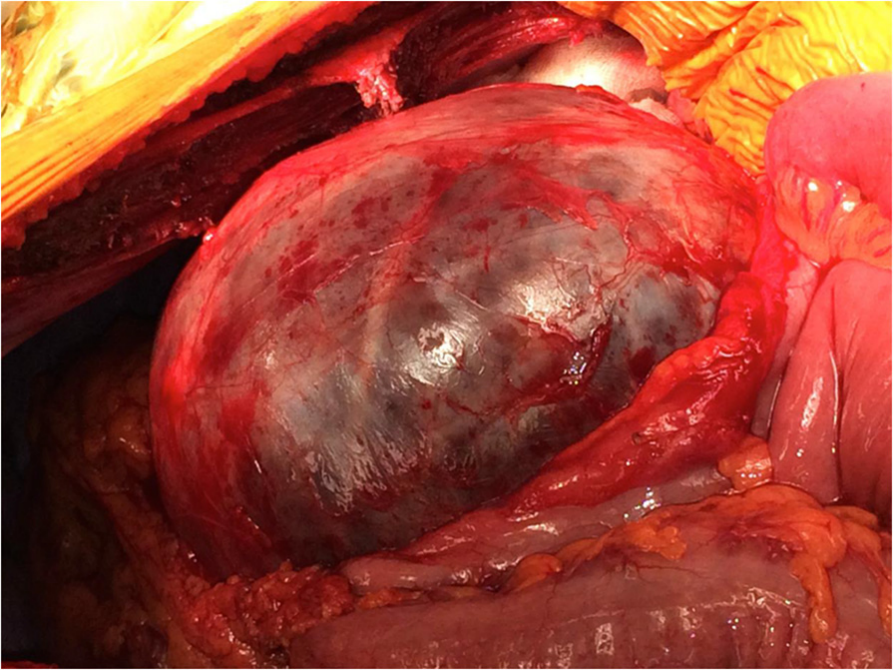
**Operative image of retroperitoneal cyst.**

**Figure 3:**
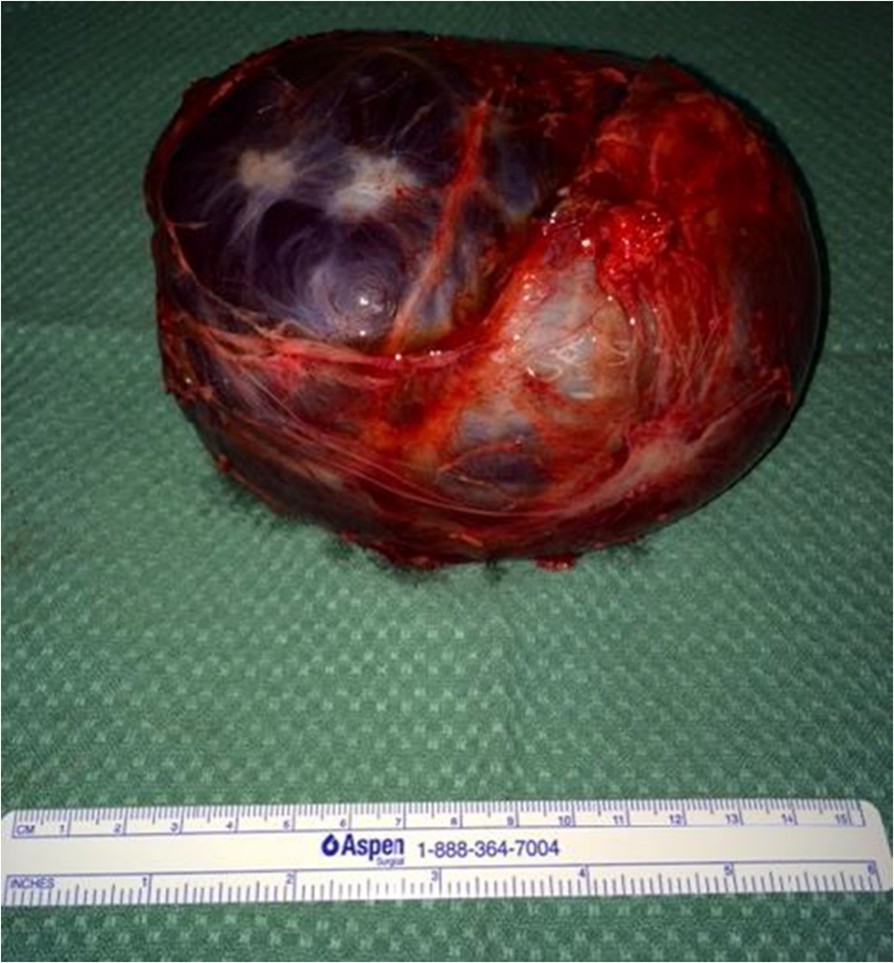
**Resected retroperitoneal cyst.**

After discussion with the patient, the decision was made to resect the cyst due to his increasing symptoms of abdominal pain and for diagnostic certainty. At operation, the cyst was isolated from the pancreas and dissected from the left ureter, left kidney, renal vessels and aorta and did not communicate with any of these structures (Figures 2 and 3). The spleen was preserved. His recovery post-operatively was uneventful. His post-prandial pain and vomiting resolved and he was able to eat better post-operatively.

Histopathological assessment of the resected cyst revealed no epithelial lining to this cyst, and it was in fact a large fibrous pseudocyst.

As the cyst was delivered intact for pathological assessment, the fluid was not tested for amylase, or lipase.

## Discussion

Our patient had a large pseudocyst in the retroperitoneum, which developed in the absence of a history of pancreatitis. Non-pancreatic pseudocysts are rare lesions which often arise from the mesentery or omentum [2]. They characteristically have a thick, fibrous wall lacking an epithelial lining, and contain haemorrhage, pus or serous fluid, which unlike pancreatic pseudocysts is not associated with high levels of amylase and lipase. On imaging they present as unilocular or multilocular cystic masses with thick walls [3]. They can develop secondary to trauma or infection and are thought to be the sequelae of a mesenteric or omental haematoma or an abscess which was not resorbed [5,6].

A RPC includes a wide differential such as mesenteric, omental, splenic and enteric duplication cysts [2]. Neoplastic RPCs can be divided into epithelial (mucinous or serous cystadenoma), mesothelial (mesothelioma), germ cell (cystic teratoma) and cystic changes in a solid neoplasm (paraganglioma, neurilemmoma, sarcoma) [2]. Other rare cysts such as lymphangioma (1 % of all retroperitoneal neoplasms [7]), mullerian cyst, epidermoid cyst, tailgut cyst, bronchogenic cyst, pseudomyxoma retroperitonei, perianal mucinous cystadenoma have been described. Non-neoplastic causes include haematoma, urinoma, lymphocele, pancreatic pseudocyst and nonpancreatic pseudocyst [7].

Diagnostic work-up should include a thorough history, laboratory investigations including tumour markers and imaging. Assessment of the retroperitoneum with a CT will allow characterisation of the cystic mass and its relationship with surrounding organs. Magnetic resonance imaging (MRI) was used in this instance, as MRI has superior contrast resolution enabling soft-tissue delineation and is useful in characterising cysts by depicting septae, ductal communications, wall thickening and enhancement [2,8].

Many cystic lesions cannot be accurately diagnosed as clearly benign without operative and pathological evaluation. The decision to proceed to surgery is determined by the presence of symptoms, risk of complications without intervention (infection, rupture, possibility of malignant change) and diagnostic uncertainty [9].

In this case we were unable to assess the cyst fluid for amylase, as the cyst was sent for pathological assessment intact, and a pancreatic pseudocyst was low in our differentials, given the patient gave no history of pancreatitis and there was no communication with the pancreas.

Pseudocysts can be complicated by infection, haemorrhage and mass effects [9,10]. Management options include simple drainage and complete excision by laparotomy, an extraperitoneal approach, transperitoneal flank approach or laparoscopic excision. Complete excision is preferred to avoid potential for recurrence [2,11].

## Conclusions

We present a rare case of an isolated large retroperitoneal fibrous pseudocyst unrelated to previous pancreatitis which was successfully managed with surgery.

## Consent

Written informed consent was obtained from the patient for publication of this Case report and any accompanying images. A copy of the written consent is available for review by the Editor of this journal.

## References

[1] Izaraa A, Mousa H, Dickens P, Allen J, Benhamida A (2008). Idiopathic benign retroperitoneal cyst: a case report. J Med Case Rep.

[2] Yang DM, Jung DH, Kim H, Kang JH, Kim SH, Kim JH (2004). Retroperitoneal cystic masses: CT, clinical and pathological findings and literature review. Radiographics.

[3] Rajiah P, Sinha R, Cuevas C, Dubinsky T, Bush W, Kolokythas O (2011). Imaging of uncommon retroperitoneal masses. Radiographics.

[4] Kim KO, Kim TN (2012). Acute pancreatic pseudocyst: incidence, risk factors and clinical outcomes. Pancreas.

[5] Cizginer S, Tatli S, Snyder E, Goldberg J, Silverman S (2009). CT and MRI imaging features of a non-pancreatic pseudocyst of the mesentery. European J Gen Med.

[6] Stoupis C, Ros P, Abbitt P, Burton S, Gauger J (1994). Bubbles in the belly: imaging of cystic mesenteric or omental masses. Radiographics.

[7] Nishino M, Hayakawa K, Minami M, Yamamoto A, Ueda H, Takasu K (2003). Primary retroperitoneal neoplasms: CT and MR findings with anatomic and pathologic diagnostic clues. Radiographics.

[8] Renzulli P, Candidas D (2009). Symptomatic retroperitoneal cyst: a diagnostic challenge. Ann R Coll Surg Engl.

[9] Tanaka M, Castillo C, Adsay V, Chari S, Falconi M, Jang JY (2012). International consensus guidelines 2012 for the management of IPMN and MCN of the pancreas. Pancreatology.

[10] Oray-Schrom P, Martin D, Bartelloni P, Amoateng-Adjepong Y (2002). Giant nonpancreatic pseudocyst causing acute anuria. J Clin Gastroenterol.

[11] Geng JH, Huang CH, Wu WJ, Huang SP, Chen YT (2012). Huge retroperitoneal nonpancreatic pseudocyst. Urological Sci.

